# Bridging Solution and Solid-State Mechanism: Confined Quasi-Solid-State Conversion in Li–S Batteries

**DOI:** 10.1021/acsenergylett.5c02093

**Published:** 2025-10-25

**Authors:** Pronoy Dutta, Jean-Marc von Mentlen, Soumyadip Mondal, Nikolaos Kostoglou, Bodo D. Wilts, Stefan A. Freunberger, Gregor A. Zickler, Christian Prehal

**Affiliations:** † Department of Chemistry and Physics of Materials, 625366 University of Salzburg, Jakob-Haringer-Straße 2A, 5020 Salzburg, Austria; ‡ Department of Information Technology and Electrical Engineering, ETH Zürich, Gloriastrasse 35, 8092 Zürich, Switzerland; § 148492 Institute of Science and Technology Austria, Am Campus 1, 3400 Klosterneuburg, Austria; ∥ Department of Materials Science, 27268 Montanuniversität Leoben, Franz-Josef-Straße 18, 8700 Leoben, Austria; ⊥ Institute of Geoenergy, Foundation for Research and Technology - Hellas, 73100 Chania, Greece

## Abstract

“Quasi-solid-state” conversion mechanisms using sparingly solvating electrolytes (SPSEs) bridge the gap between traditional solid–liquid–solid and solid-state sulfur conversion in lithium–sulfur (Li–S) batteries. Although these terms are commonly used, their precise distinctions and impacts on key performance metrics, such as rate capability, energy density, and capacity fading, remain poorly understood. In this work, we employ operando small- and wide-angle X-ray scattering alongside cryogenic transmission electron microscopy (cryo-TEM) to compare Li–S batteries in sparingly solvating and solvating ether-based electrolytes. We find that, unlike solvating electrolytes, SPSEs lead to an extended presence of lithium sulfide during cycling, coexisting with sulfur at a 50% state of charge and beyond. In the charged state, solid sulfur is present in its amorphous form inside the carbon black nanopores. These findings indicate that the limited solubility confines polysulfides in regions near the carbon surface, where these polysulfides enable conversion between the coexisting solid discharge and charge product.

## Graphical abstract

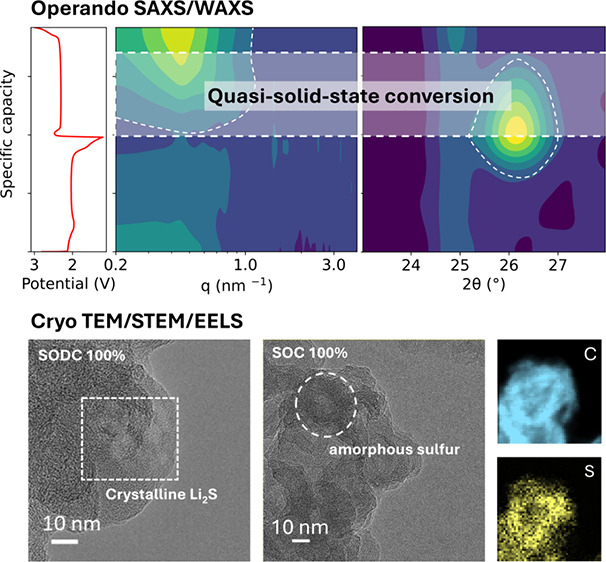

Lithium-sulfur (Li–S) batteries are a promising next-generation energy storage technology, offering high performance, low cost, excellent safety, and abundance of natural resources.^1–4^ Sulfur as an active material offers an exceptionally high theoretical specific capacity (1675 mAh g^–1^) and energy density (2500 Wh kg^–1^).^5–7^ Additionally, sulfur cathodes enable the use of low-cost, lightweight carbon materials as cathodes rather than resource-limited metal oxides. This makes the aspirational cell-level energy density goals (500 Wh kg^–1^) a tangible prospect.^8,9^ However, the fundamental sulfur-to-sulfide conversion remains insufficiently understood, especially under lean electrolyte conditions, resulting in performance limitations tied to polysulfide shuttling, capacity fade, and insufficient active material fractions.^10–14^

The primary challenge in Li–S batteries arises from the nonconductive nature of charged and discharged products, i.e., sulfur and lithium sulfide (Li_2_S), which inevitably impedes electron transfer processes during discharging and charging.^15,16^ Currently, three different reaction pathways are distinguished in liquid-electrolyte Li–S batteries: a) dissolution and precipitation (solid–liquid–solid) mechanism in standard solvating ether-based electrolytes;^17–19^ b) solid-state conversion, for example, in the confinement of microporous carbons using carbonate electrolytes;^20–23^ and c) quasi-solid-state (QSS) conversion mechanism using sparingly solvating electrolytes (SPSEs).^24–29^

While conventional ether-based liquid electrolytes rely on a solid–liquid–solid formation involving polysulfides (Li_2_S_*x*_, *x* = 2–8) to help facilitate faster ionic and electronic conductivity, their high polysulfide solubility presents critical challenges.^30^ In addition to accelerating capacity fading via the shuttle effect, the high solubility fundamentally obstructs the path toward high energy densities. As the electrolyte-to-sulfur (E/S) ratio is lowered to improve energy density, polysulfide saturation leads to gelation, a physical state that severely inhibits ion transport.^31,32^ Electrolytes and lithium salts get closely intertwined with polysulfides, leaving little to no free solvent molecules, which leads to high overpotentials and, ultimately, cell failure.^33^ As such, the use of solvating electrolytes fundamentally limits the practical implementation of lean-electrolyte, high-loading Li–S batteries.

Conversely, solid-state and quasi-solid-state sulfur conversions offer a thermodynamic route to lean-electrolyte operation without failure. By suppressing polysulfide solubility, QSS with SPSEs entirely avoids gelation and, in theory, allows for stable operation at ultralow E/S ratios.^34^ Solid sulfur, solid Li_2_S, and polysulfides dissolved at ultralow concentrations in the electrolyte can coexist, regardless of the E/S ratio, paving the way for high-energy-density Li–S batteries.^35–37^ This not only reduces shuttle effects and parasitic reactions typical of liquid systems but also improves ion transport and kinetic limitations compared to fully solid-state counterparts.^38–42^ Nazar et al. first demonstrated this possibility with a specially designed SPSE, where the two-plateau discharge behavior was reduced to a single plateau discharge, closely resembling solid-state conversion.^43^ Although these works show favorable properties and provide fundamental insights into the thermodynamics of QSS batteries, many aspects during electrochemical cycling in a working cell remain unresolved. It is unclear how polysulfide intermediates, confined to regions at the carbon-electrolyte interface, mediate conversion, and whether Li_2_S and sulfur coexist during the reaction process, an outcome incompatible with standard dissolution-preparation mechanisms.

In this study, we first employ operando small- and wide-angle X-ray scattering (SAXS/WAXS) to investigate the nanoscale structural evolution of sulfur and during cycling in Li–S batteries using sparingly solvating and standard solvating ether-based electrolytes. Subsequently, we use ex situ cryogenic transmission electron microscopy (cryo-TEM) and electron energy loss spectroscopy (EELS) to confirm the spatial distribution and phase composition of discharge and charge products. Our results reveal a distinct reaction pathway highlighting the extended coexistence of nanocrystalline Li_2_S and solid sulfur during cycling, which significantly diverges from the classical dissolution–precipitation model. This work establishes a framework to understand confined solid-phase reactions and their role in enabling lean-electrolyte high-energy Li–S battery systems.

To compare solid–liquid–solid and QSS conversion, we use a common solvating ether-based electrolyte (1 M lithium bis­(trifluoro­methane)­sulfonimide, LiTFSI, in dioxolane and dimethoxyethane, DOL/DME, v/v 1:3) and a SPSE consisting of 6 M LiTFSI in DME diluted with a nonsolvent hydrofluoroether (HFE, v/v 1:2). These electrolytes were combined with a high-surface-area carbon black cathode (Ketjenblack, KB, 1400 m^2^ g^–1^) for all the electrochemical investigations. The galvanostatic discharge–charge curves for the SPSE exhibit a single, flat discharge plateau at ∼2.1 V and a charging plateau at ∼2.3 V, distinctly different from the solvating ether electrolyte, which shows a two-plateau discharge profile (2.4 and 2.1 V, Figure S1). The SPSE, however, shows a noticeable dip in potential around 300 mAh g^–1^ during discharge, which is also mirrored at the onset of charging. This feature exhibits nonideal QSS behavior (i.e., a perfect single plateau) and raises the question of whether these deviations are driven by kinetic limitations or underlying thermodynamics. To clarify this, galvanostatic intermittent titration technique (GITT) measurements were performed on both electrolyte systems. The quasi-equilibrium potential derived from GITT reveals that the solvating ether electrolyte maintains two characteristic plateaus during both discharge and charge (Figure 1a). In contrast, the SPSE system exhibits a nearly constant relaxation potential throughout the entire discharge and charge process, at ∼2.2 and 2.25 V, respectively (Figure 1b). Interestingly, the transient humps and dips in the SPSE voltage profile correspond to quasi-equilibrium values aligned with the rest of the discharge–charge curve, suggesting that these deviations are kinetic in origin, likely due to nucleation barriers associated with Li_2_S formation rather than phase transition events.

**Figure 1:**
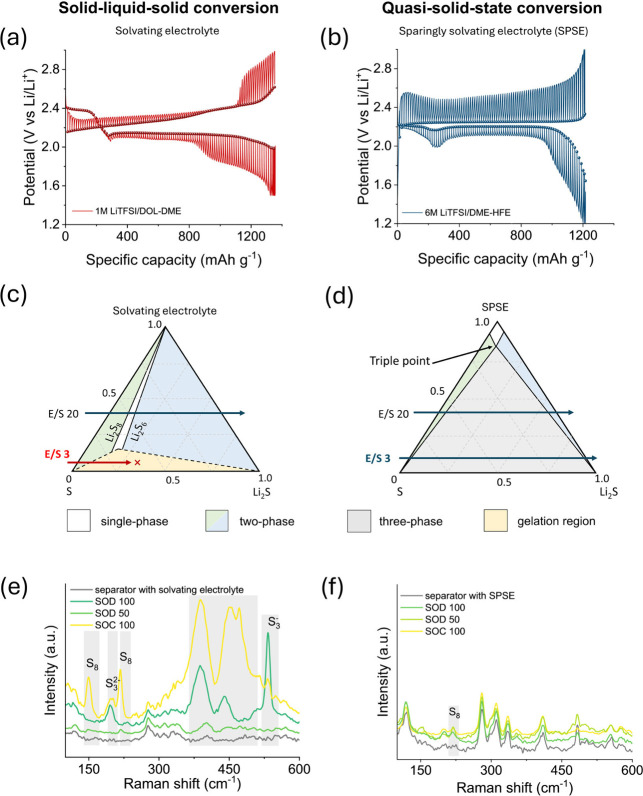
Distinguishing solid–liquid–solid from quasi-solid-state sulfur to sulfide conversion. GITT measurements showing the quasi-equilibrium potentials during discharging/charging for (a) the solvating electrolyte and (b) the SPSE electrolyte system. The two distinctly different plateau potentials during discharge in the solvating ether electrolyte indicate the presence of two different thermodynamic equilibria corresponding to the solid–liquid–solid conversion process, whereas the constant potential response for the SPSE suggests a single persistent equilibrium throughout discharge. Theoretical phase diagrams indicating the phase equilibrium between sulfur, Li_2_S, and dissolved polysulfides for (c) solvating ether-based electrolyte system, and (d) SPSE electrolytes in Li–S batteries. The horizontal line across the phase diagram corresponds to a certain E/S ratio in the system. Comparative Raman spectra of (e) standard solvating system, and (f) SPSE at different SOD/SOCs. The gray-highlighted regions indicate changes in vibrational features associated with different electrochemical states. Note that the background reference spectra of the separator with the bare electrolyte and without polysulfides are given in gray.

To further contextualize the electrochemical behavior of solid–liquid–solid and QSS sulfur conversion, we introduce theoretical ternary phase diagrams, which map the phase stability of sulfur, Li_2_S, and polysulfides across different E/S ratios and states of charge (SOCs).^35^ Figure 1c represents a typical phase diagram of a standard solvating electrolyte system consisting of three distinct phase regions: single-phase, two-phase, and a gelation region at the bottom, which corresponds to lean electrolyte conditions at low E/S ratios. As a system with a specific E/S ratio follows a discharge trajectory from left to right (indicated by the horizontal lines across the diagram), it traverses the phase boundaries, resulting in the characteristic voltage profile (Figure S2). When the system moves through the first two-phase region (S – Li_2_S_8_), the potential remains constant, resulting in a plateau. For the following single-phase region (Li_2_S_*x*_, 6 < *x* < 8), the electrode potential decreases as the state of discharge (SOD) evolves (slope region). Together with the following two-phase region (Li_2_S_6_ – Li_2_S), this translates to the characteristic two-plateau voltage profile in solvating electrolytes.^5^ However, as the E/S ratio goes below a certain threshold, the electrolyte becomes saturated with polysulfides, leading to long-range cross-linking and gelation. Electrochemical operation of Li–S systems under such conditions results in high overpotentials, which eventually leads to complete cell failure.^31,44–46^

Conversely, for SPSEs with decreased polysulfide solubility, the system shows a triple-point at high E/S ratios, leading to an extended three-phase region of sulfur, Li_2_S, and electrolyte with low polysulfide concentration (Figure 1d). In principle, an extremely low E/S ratio can now be realized without gelation, avoiding substantial overpotentials or cell failures. As the cathode primarily resides in this three-phase region during discharge, we expect a constant potential throughout the process, much like a solid-state conversion (Figure S3). The GITT data in Figure 1b confirm the constant equilibrium potential, indicating that SPSEs promote a QSS mechanism governed by triple-phase equilibrium, in contrast to the dissolution–precipitation behavior of solvating electrolyte systems.

To experimentally validate the proposed triple-phase equilibrium, we examined polysulfide speciation in separators retrieved at different SODs and SOCs using visual inspection, UV–vis, and Raman spectroscopy. In the solvating electrolyte, separators show pronounced color changes and evolving spectroscopic signatures consistent with the progressive reduction of long-chain to shorter-chain polysulfides, reflecting the classical dissolution–precipitation pathway.^47–51^ Distinct Raman peaks evolve with cycling, including bands at 150 cm^–1^ and 217 cm^–1^ (S_8_) at 100% SOC and a strong S_3_^–^ signal at 532 cm^–1^ at 100% SOD (Figure 1e).^17,50,52^ The pronounced peaks evolving between 350 and 480 cm^–1^ have also been observed in other Raman studies,^17,50,52^ but cannot be uniquely assigned to a certain polysulfide. In contrast, separators tested in SPSE cells remain uniformly whitish to pale yellow, with UV–vis spectra suggesting the dominance of short-chain species and Raman spectra nearly identical to the pristine electrolyte-soaked reference (Figures 1f, S4, S5).^47,49^ This further confirms the minimal crossover and confined speciation associated with the QSS conversion pathway. Collectively, these results demonstrate that soluble polysulfide formation is strongly suppressed and that the cathode remains confined to a QSS three-phase regime throughout cycling (discussed in detail in Figures S4–S5).

Operando SAXS/WAXS was also performed with identical KB/S cathodes with standard solvating electrolyte (1 M LiTFSI in diglyme) and SPSE (6 M LiTFSI in DME-HFE) to gain detailed insights into atomic and nanoscale structural evaluation during electrochemical cycling. Our setup ensures that we probe structural changes in the KB/S cathode. For the solvating electrolyte, the SAXS intensity variation during discharge is shown in the double-logarithmic plots in Figure 2a, with the scattering vector *q* ranging between 0.2 and 4 nm^–1^. As sulfur converts to Li_2_S and back during discharging and charging, the X-ray scattering intensity of the cathode exhibits varying intensity across different *q*-range segments, indicating a length-scale-dependent structure evolution. The SAXS scattering intensity in the high *q* region (0.9–3 nm^–1^), increases as we discharge, whereas the intensity goes down almost reversibly during charging (Figure S6). The WAXS intensity in Figure 2b shows the evolution of the Li_2_S (111) diffraction peak during discharge at ∼42% SOD, while the crystalline sulfur diffraction peaks disappear.

**Figure 2:**
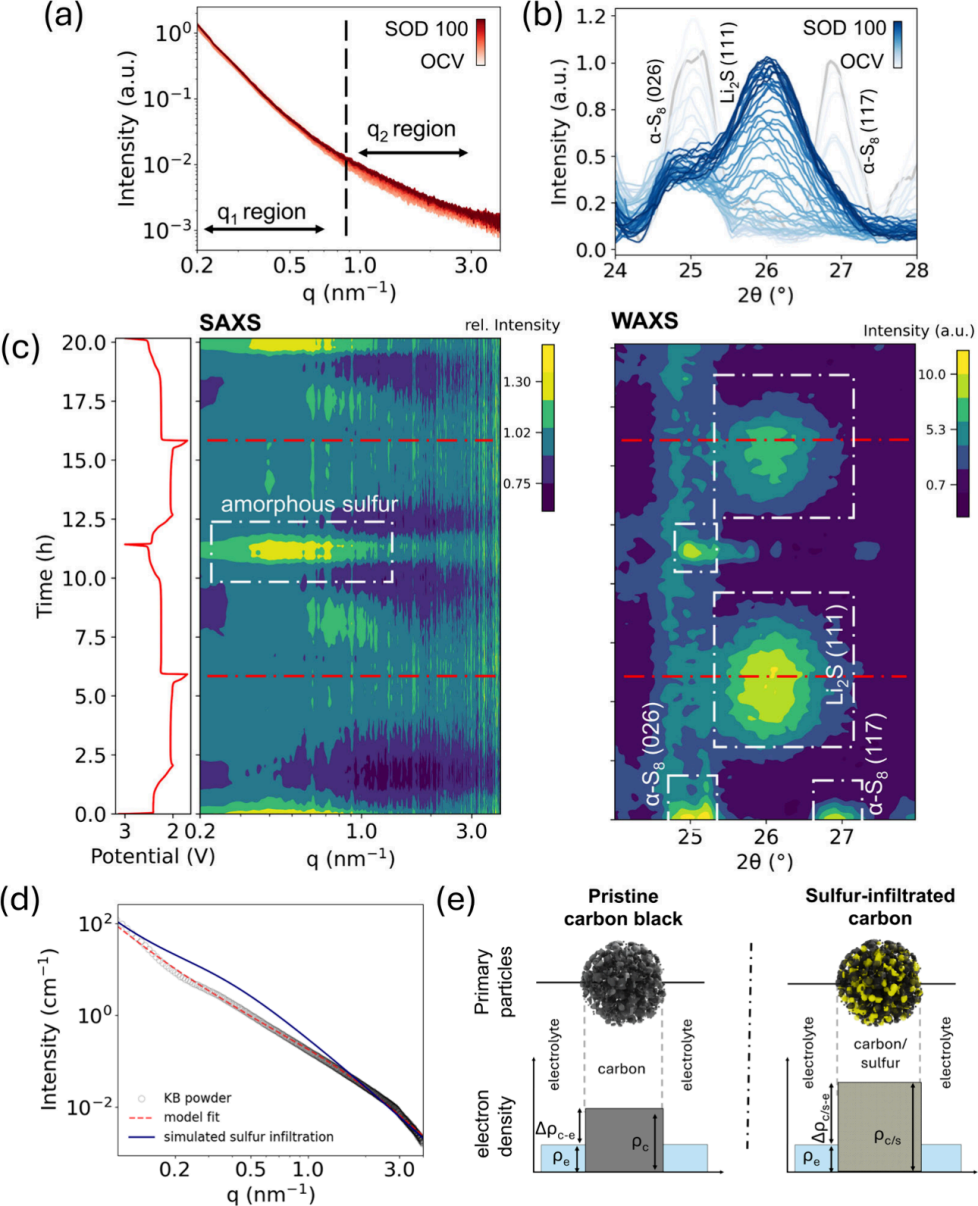
Operando SAXS/WAXS measurements of a Li–S cell with solvating electrolyte. (a) The SAXS intensity response of KB cathodes during galvanostatic discharge at C/10 rates; (b) the corresponding WAXS response with disappearing sulfur peaks as the discharge progresses. The time-resolved operando (c) SAXS (left), and WAXS (right) intensity maps w.r.t. scattering vector *q* and time of cycling, highlighting the time evolution of sulfur and Li_2_S during electrochemical processes. The red lines across the diagrams indicate the end of the discharge cycle. (d) Background corrected SAXS intensity versus scattering vector *q* plot with corresponding model fit in red. The blue profile corresponds to a simulated SAXS curve at the end of charging, where the pores of the KB primary particles are filled with sulfur. (e) Schematic illustration of the electron density differences as the sulfur moves in and out of the pores of KB particles. With sulfur infiltration, the overall electron density of the primary carbon black particles increases significantly, thus increasing the SAXS intensity response during charging.

To highlight the SAXS intensity response during two consecutive discharge/charge cycles, the relative SAXS intensity (normalized to the curve at full discharge) is plotted as a function of time and scattering vector *q* in a contour plot (Figure 2c, left). The corresponding WAXS intensity evolution is given on the right as a function of the scattering angle 2θ. The SAXS contour map highlights two separate regions of activity, *q*_1_ (0.2–0.7 nm^–1^) and *q*_2_ (0.9 to 3 nm^–1^). Notably, at the end of charging, a pronounced intensity maximum appears in the *q*_1_ region, corresponding to a characteristic feature size of (2π/*q*) ∼ 21 nm. As this dimension matches the primary particle size of carbon black (KB), we attribute the intensity maximum to the formation of amorphous sulfur within the nanopores of the KB particles.

To verify this, the SAXS intensity profile of the pristine KB powder was fitted using a model describing the porous aggregate structure, including scattering contributions from the fractal-like KB particle aggregates and the internal nanopores (details in Supporting Note 1). Modifying the scattering length density (SLD) and internal nanopore volume fraction to simulate ∼80% pore filling with sulfur resulted in a distinct hump emerging in the simulated SAXS intensity curve around the same *q*_1_ region (Figure 2d). Conceptually illustrated in Figure 2e, sulfur infiltration increases the overall electron density contrast of the KB particle with respect to the surrounding medium. Since the SAXS intensity scales with the square of the electron density difference (*I*∝ Δρ^2^), pore filling leads to the observed intensity increase at low *q*. Please note that independent verification of the SAXS intensity maximum at 100% SOC is provided further below using cryo-STEM-EELS.

In WAXS, crystalline sulfur is observed at 25° and 27° at the beginning, corresponding to α-S (026) and (117), respectively. At full discharge, sulfur reflections disappear, and only the Li_2_S (111) peak remains, confirming complete conversion. At 100% SOC, a weak sulfur diffraction peak emerges. Given the high sulfur loading (66 wt %) and the low intensities of these peaks, we conclude that only a small fraction of sulfur recrystallizes at external surfaces, while a significant fraction remains amorphous and confined within the pores of KB, as seen by SAXS (Figure 2c-e).

Having established the separate SAXS and WAXS responses, it is instructive to compare their evolution directly. At the beginning of discharge, the SAXS intensity in the *q*_2_ region decreases, before it increases again to form a weak intensity maximum at the end of discharge. The simultaneous formation of nanocrystalline Li_2_S in WAXS (diffraction peaks at 26° in Figure 2c) and the concurrent intensity response at *q*_2_ in SAXS do not reflect the same particle sizes (Supporting Note 2, Figure S7, ∼7 nm for Li_2_S from WAXS versus ∼4 nm at discharge estimated from the SAXS intensity maximum). Furthermore, at the onset of charging_,_ the *q*_2_ intensity maximum shifts toward lower *q* (Supporting Note 2), indicating that more than one discharge product is involved. This finding is in line with our previous small-angle neutron studies, which provided evidence for a composite discharge product, consisting of nanocrystalline Li_2_S embedded in an amorphous Li_2_S_2_ phase with feature sizes around 3–4 nm.^17,53^

Operando SAXS/WAXS measurements on the SPSE cell reveal distinct differences in the electrochemical conversion mechanism compared to that of the solvating electrolyte system. The SAXS and WAXS intensity profiles during the first discharge are shown in Figure 3a, b, while the corresponding time and *q*-dependent intensity changes over two cycles are visualized in the SAXS/WAXS contour plots in Figure 3c. In the SAXS response, the high-*q* region (*q*_2_) remains largely unaffected throughout cycling, showing no significant pattern formation. By contrast, the low-*q* region (*q*_1_), exhibits pronounced intensity changes. The intensity decreases during discharge and increases during charge, developing a clear maximum that becomes particularly evident from the first charging cycle (Figure 3c and Figure S8). This *q*_1_ maximum corresponds to amorphous sulfur and, in the SPSE system, appears significantly earlier during charging than in the solvating electrolyte (Figure 3c). Amorphous sulfur dissolution becomes slower during the second discharge, possibly due to the optimized restructuring of amorphous sulfur within the nanopore confinement of the KB particles after the first complete cycle.

**Figure 3:**
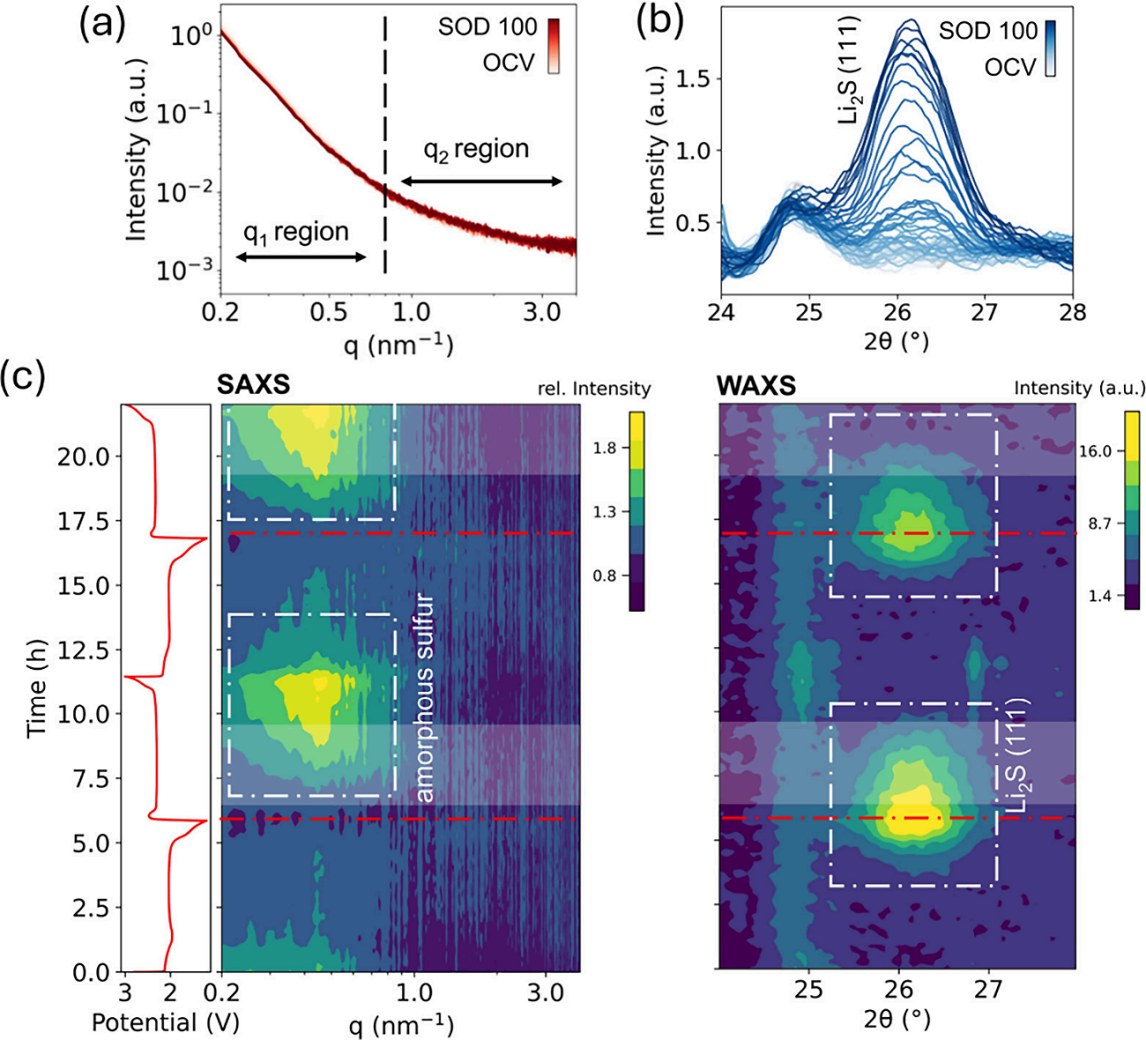
Operando SAXS/WAXS measurements of a Li–S cell with SPSE. (a) The SAXS intensity response of KB cathodes during galvanostatic discharge at C/10 rates; (b) the corresponding WAXS response with increasing Li_2_S (111) scattering as the discharge progresses. The time-resolved operando (c) SAXS and (d) WAXS intensity maps w.r.t. scattering vector *q* and specific capacity highlighting the time evolution of sulfur and Li_2_S during the electrochemical processes. The red lines across the diagrams indicate the end of the discharge cycle. The highlighted white strips show the overlap of sulfur and Li_2_S during the charging process.

The WAXS response complements these observations. The crystalline Li_2_S (111) diffraction peak emerges at ∼48% SOD during discharge and persists until ∼86% SOC upon charging, coexisting with the *q*_1_ sulfur maximum in SAXS, which begins forming already at ∼23% SOC. This contrasts sharply with the solvating electrolyte, where sulfur evolves only at ∼80% SOC, once Li_2_S consumption is complete. Moreover, crystalline sulfur formation is strongly suppressed in SPSEs, as demonstrated by the negligible intensities of S diffraction peaks near 25° and 26.7° at the end of the first charge.

Importantly, the SAXS/WAXS intensity response associated with amorphous sulfur and crystalline Li_2_S is not entirely symmetric between the discharge and charge. Hence, conversion in the real SPSE system deviates from the equilibrium behavior predicted from phase diagrams.

The contrast between the two conversion mechanisms becomes even clearer when analyzing the time evolution of the integrated intensity of the SAXS *q*_1_ region (indicative for sulfur formation and dissolution) and the area under the Li_2_S (111) diffraction peak from WAXS (using Lorentzian peak fitting), as shown in Figure 4a,b. In the solvating system, the *q*_1_ SAXS intensity quickly drops to zero by the end of the first discharge plateau, which, according to the ternary phase diagram (Figure 1c), marks the end of the two-phase region where sulfur coexists with dissolved Li_2_S_8_. The slight increase in the *q*_1_ SAXS intensity at the end of discharge is attributed to the formation of Li_2_S/Li_2_S_2_.^17,53^ The rise of the Li_2_S WAXS peak at the onset of the second discharge plateau aligns with the predicted coexistence of Li_2_S and polysulfides as the system moves into the second two-phase region. Importantly, sulfur dissolution and Li_2_S formation do not overlap, reflecting the solid–liquid–solid conversion mechanism, where Li_2_S only forms once all of the sulfur is converted into polysulfides.

**Figure 4:**
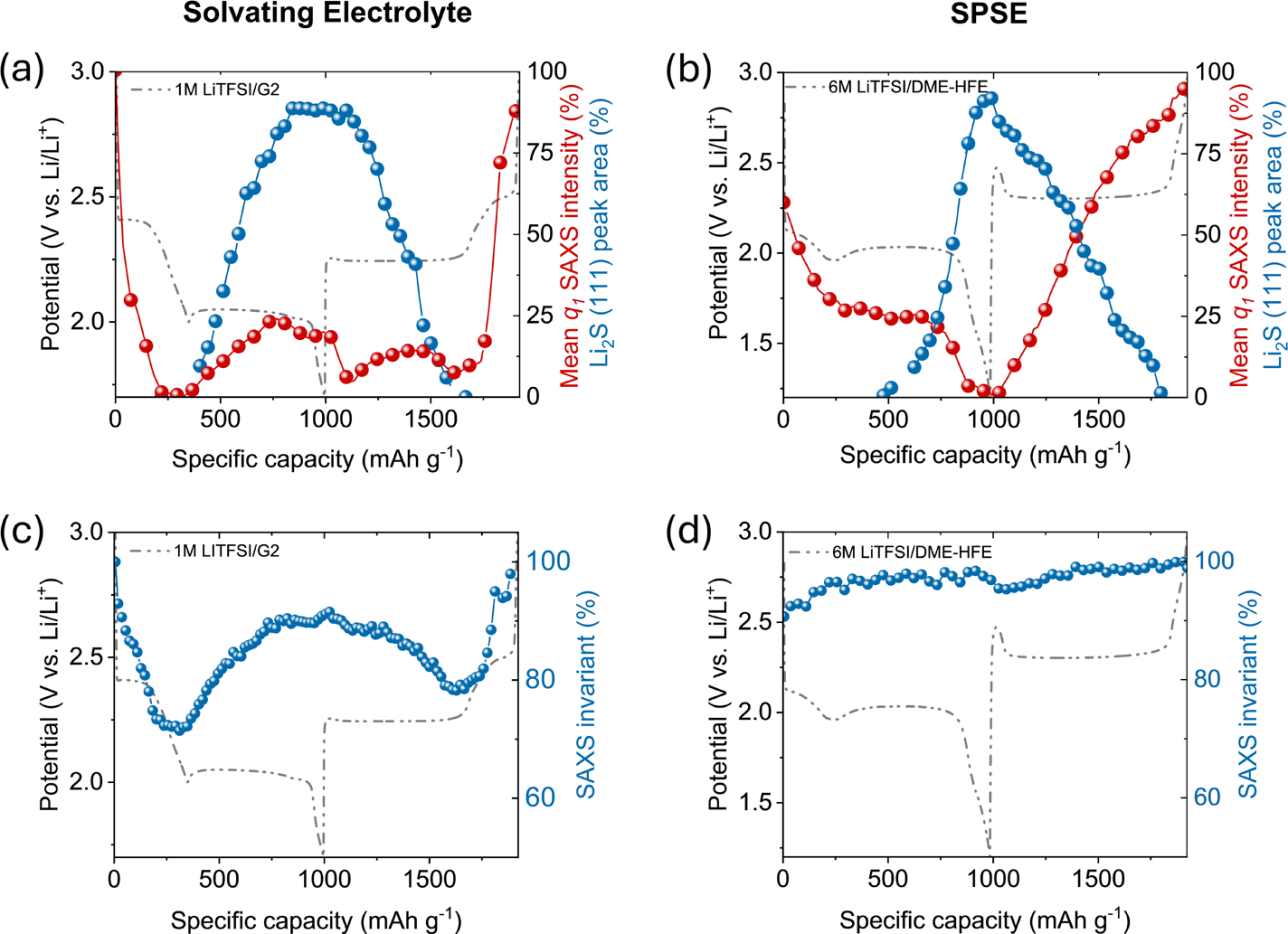
SAXS/WAXS integral parameter analysis of the second discharge/charge using the solvating electrolyte and SPSE. The change in average SAXS intensity contribution and integrated area under the Li_2_S (111) Lorentzian peak fittings from WAXS w.r.t. specific cell capacity for (a) solvating and (b) SPSE electrolyte cells. In the solvating electrolyte, the sudden increase in average intensity around 400 and 1200 mAh g^–1^ corresponds to the formation of Li_2_S_2_ in the system. The nonoverlapping behavior of discharge–charge species in the solvating electrolyte system significantly changes for SPSE as sulfur and Li_2_S coexist prominently, especially during charging. Relative change in scattering invariant (Q̃) with specific capacity for solvating electrolyte in (c) and for SPSE in (d). The variation in Q̃ is significant for the solvating electrolyte, but stays relatively constant for the SPSE system.

In contrast, the SPSE system shows a clear coexistence of solid sulfur and solid Li_2_S during discharge, along with a pronounced “cross” pattern during charge (Figure 4b), indicating strong coupling between Li_2_S consumption and simultaneous sulfur formation. This coexistence of solid discharge and charge products is in line with the equilibrium phase behavior of the QSS conversion (Figure 1d).

During discharge and charge, the dissolution and migration of polysulfides into the electrolyte alter the electron density contrast between the cathode and the electrolyte. To track this process, we analyzed the scattering invariant (Q̃) over the entire *q* range (0.2–4 nm^–1^), which reflects changes in electron density contrast and the volume fractions of phases within the irradiated volume.^55^ A variation in Q̃ indicates that species are leaving or entering the irradiated volume at the SAXS sensitive length scales (2π/0.2 nm^–1^–2π/4.0 nm^–1^).^54^

In the solvating electrolyte (Figure 4c), Q̃ gradually decreases at the onset of discharge and drops by nearly 30% by the end of the first discharge plateau (∼340 mAh g^–1^), where all sulfur is expected to be dissolved as polysulfides. This reduction arises from polysulfide diffusion into the electrolyte outside the irradiated volume, lowering the electron density contrast at the solid-electrolyte interface, and is consistent with the separator discoloration observed in Figure S4. As discharge continues and solid Li_2_S nucleates and precipitates in the cathode, consistent with a dissolution–precipitation mechanism, a rise in the level of Q̃ occurs. During subsequent charging, the conversion of Li_2_S into soluble polysulfides reverses this process, yielding a nearly symmetric Q̃ profile for discharge and charge.

By contrast, the SPSE system exhibits a relatively constant Q̃ during both discharge and charge, with small changes only at the initial stages of each process (Figure 4d). This stability suggests that active species remain confined within the cathode, close to the carbon-electrolyte interface, preventing major density contrast changes. Combined with the coexisting presence of Li_2_S and amorphous S, this strongly supports a solid–solid mechanism in SPSEs. Here, a low concentration of soluble polysulfides serves as a transient mediator, localized to regions close to the carbon-electrolyte interface. This is distinct from the solid–liquid–solid conversion in standard solvating electrolytes. The suppressed Q̃ variation reinforces the limited involvement of bulk solution pathways in the QSS conversion reaction.

The confinement of polysulfides to regions close to the carbon-electrolyte interface also translates into significantly improved cycling stability in SPSE cells. At a C/10 rate over 38 cycles, the SPSE-based cell retained 90% of its initial capacity compared to only 28% for the solvating electrolyte (Figure S9a). The SPSE separator recovered from the cell after cycling showed no visible discoloration, whereas the solvating electrolyte separator was heavily stained (Figures S9b,c). Raman spectra further confirm this contrast; the SPSE separators remain identical to the uncycled reference, while the solvating electrolyte separators exhibited strong signatures of accumulated polysulfides (Figures S9d).

Operando scattering data offer unique, time-resolved, ensemble-averaged insights into structural evolution; however, their interpretation often relies on prior knowledge. Local model-free structural and chemical information obtained from cryo-TEM and EELS, therefore, ideally complements the operando SAXS/WAXS results for the SPSE electrolyte. Here, two different approaches were taken to overcome the severe experimental challenges of beam damage, air contamination, and low-pressure evaporation during TEM on discharged and charged Li–S battery cathodes (details, see SI).^56^ Briefly, a binder-free cathode consisting of melt-infiltrated C/S particles on a glassy carbon disc was discharged and charged to three different states: a) fully discharged, b) partially charged to 50% SOC, and c) fully charged to 100% SOC. Despite the absence of a binder, the electrochemical behavior closely matches that of cathodes prepared using the standard method (Figure S10). After reaching the desired DOD or SOC, the powder was transferred onto the TEM grid without washing. This protects the pristine nature of the cathode by preventing the dissolution of soluble polysulfides.^17,53^

Figure 5a shows an agglomerate of discharged particles consisting of KB and discharge products. High-resolution imaging reveals lattice fringes corresponding to crystalline Li_2_S, as confirmed by Fast-Fourier-Transform (FFT) analysis, with lattice spacings consistent with the (111) planes of Li_2_S (Figure 5b, Figure S11). In addition to the 10–30 nm crystalline domains, an amorphous region is also visible at the particle edges, which may correspond to Li_2_S_2,_ similar to observations in solvating electrolytes.^17,53^

**Figure 5:**
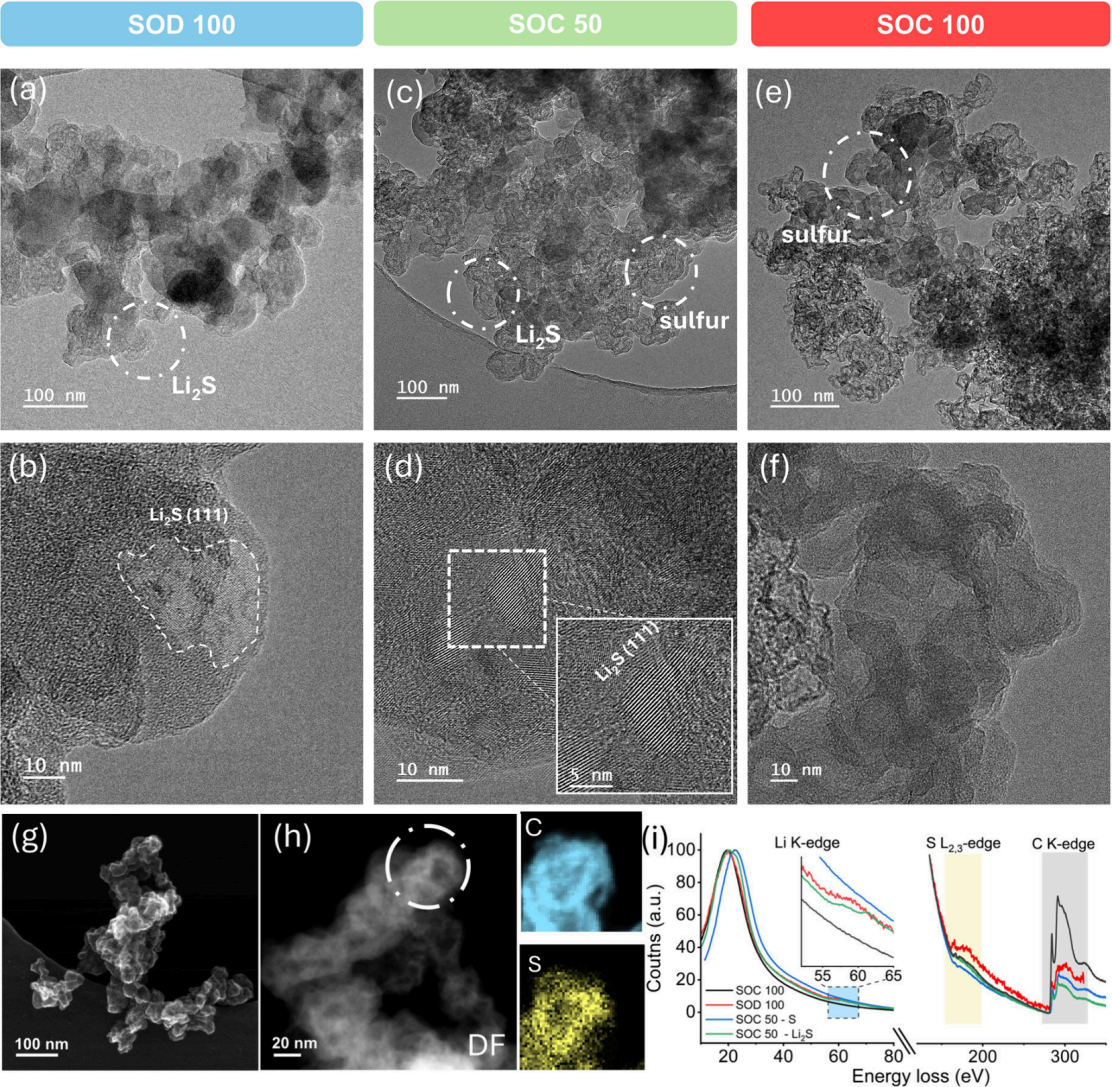
Cryo-TEM analysis of SPSE Li–S battery cathodes at different states of discharge–charge. (a) Overview of representative fully discharged (SOD 100%) KB particles with discharge products highlighted in white. (b) High-resolution image of discharge products superimposed with Fourier-filtered lattice fringes, corroborating the lattice spacing of Li_2_S (111). The crystalline regions span approximately 25–30 nm. (c) Representative image of cathode powder at 50% SOC, exhibiting two distinct regions: smooth surfaces at the particle edges showing crystalline fringes similar to those at 100% SOD (shown in panel (d)), and particles exhibiting a visible density gradient with entirely amorphous regions. Such “core–shell” features become increasingly prominent upon full charge, as shown in panel (e) with the (f) corresponding magnified image highlighting a distinct radial density gradient and the complete absence of crystallinity. (g) Secondary electron image obtained in STEM mode of the cathode powder at 100% SOC, highlighting surface topography. The particles exhibit a distinct density gradient across their diameter, resembling core–shell-like structures. This is further visualized STEM dark-field (DF) images shown in (h) with corresponding STEM-EELS elemental mapping over the region highlighted in (h). The maps show the spatial distribution of carbon (top) and sulfur (bottom) within a KB particle, revealing a composite structure where sulfur is uniformly distributed across the carbon framework. (i) TEM-EELS spectra including both low-loss and core-loss regions, acquired from cathode powders at different electrochemical states. SOD 100 and SOC 100 refer to electrodes at full discharge and full charge, respectively. SOC 50-S and SOC 50-Li_2_S represent distinct regions of the 50% SOC sample, corresponding to the smoother outer surface and rougher inner regions in Figure 5c, respectively.

At 50% SOC, two distinct morphological regions appear (Figure 5c). We observe smoother surfaces with crystalline structures, resembling those at 100% SOD, and rough spherical aggregates with an amorphous appearance in the central area (Figure 5d, S12). Upon further charging to 100% SOC, clearly distinguishable spherical structures emerge with an average particle size of ∼30 nm in diameter (Figure 5e). This size matches the average feature size estimated from the SAXS intensity maximum at the end of charge (2π/*q* ≈ 22 nm) supporting our earlier interpretation that the SAXS intensity maximum corresponds to the formation of amorphous sulfur within the nanoporous structure of the carbon black particles. Fully charged samples (Figure 5e,f) also indicate a varying radial sulfur distribution within the particles, suggesting a “core–shell” feature, which is further supported by secondary electron and magnified dark field (DF) STEM images (Figure 5g,h).

To chemically identify the discharge and charge products, STEM-EELS elemental mapping was first performed on samples at 100% SOC, where the absence of Li_2_S allows for higher beam stability and spatial resolution (Figure 5h). The resulting elemental maps show colocalization of sulfur and carbon throughout the particle volume, indicating that sulfur fills the pores of the primary KB particles rather than forming independent sulfur structures. Gas adsorption measurements support this finding. The Brunauer–Emmett–Teller (BET) surface area decreases from 1269 m^2^ g^–1^ for pristine KB to 54 m^2^ g^–1^ after sulfur infiltration, and further to 42 m^2^ g^–1^ after full charging with SPSE, consistent with sulfur redistribution inside the intraparticular nanopores (Figure S13). In line with the WAXS data, the majority of sulfur is amorphous. Thus, the SAXS intensity maximum in the *q*_1_ region reflects the overall increase in electron density of the composite KB/S particles rather than the formation of discrete crystalline or isolated amorphous sulfur domains.

Complementary TEM-EELS analyses were then performed at 100% SOD, 50% SOC, and 100% SOC to assess the average chemical composition across the entire cycle, albeit with reduced spatial resolution from the field of view. Because of the sensitivity of Li_2_S under the high electron dose required for TEM-EELS, larger apertures (10–20 μm) were used to minimize damage while maintaining representative sampling. At 100% SOD, the EELS spectrum acquired from the region shown in Figure 5b reveals a distinct Li core-loss peak (∼60 eV) alongside the sulfur core-loss edge (∼165 eV), indicating the presence of Li_2_S_*x*_ species (Figure 5i).^53^ As discussed earlier, complementary FFT lattice matching confirms the crystalline phase as Li_2_S. At 50% SOC, EELS spectra were collected from two morphologically distinct regions. The smoother-edged particle (denoted as SOC-50 Li_2_S in Figure 5i) exhibited an energy loss profile resembling that observed at 100% SOD, featuring both the lithium and sulfur core-loss edges, indicating the continued presence of Li_2_S at this intermediate stage. In contrast, spectra acquired from the rough spherical particles revealed only the sulfur core-loss edge with no detectable lithium signal. This spectral profile closely matches that observed at 100% SOC, confirming elemental sulfur in both instances. The simultaneous detection of Li_2_S and sulfur at 50% SOC demonstrates that SPSE-based systems undergo concurrent formation and consumption of solid discharge and solid charge products, reminiscent of QSS conversion.

In conclusion, our combined operando SAXS/WAXS and ex situ cryo-TEM/EELS study provides a comprehensive mechanistic picture of QSS sulfur conversion in Li–S batteries using sparingly solvating electrolytes. In contrast to the solid–liquid–solid pathway mediated by long-range polysulfide transport, we observe a distinct conversion process characterized by the coexistence of solid Li_2_S and sulfur even at intermediate states of charge. This is different from sequential species evolution and highlights a solid-intermediate-solid mechanism, in which short-lived polysulfides at an ultralow concentration act as intermediates bound to regions near the carbon-electrolyte interface rather than the bulk electrolyte (Figure 6).

**Figure 6:**
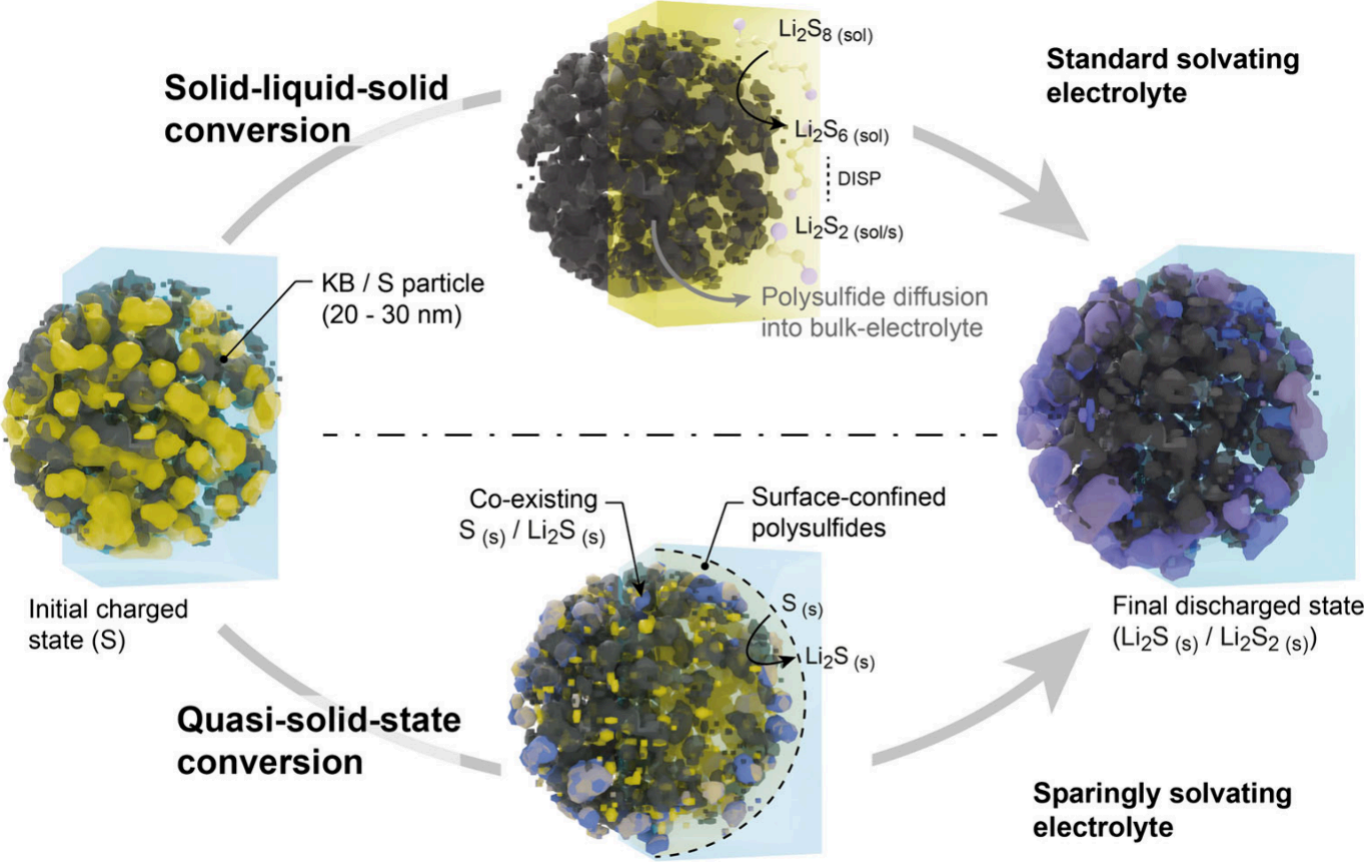
Comparative schematic of sulfur conversion pathways in solvating ether-based electrolytes versus sparingly solvating electrolytes. In ether-based systems, complete sulfur consumption occurs through bulk polysulfide dissolution prior to the formation of final discharge products (Li_2_S and Li_2_S_2_). In contrast, the limited solubility in sparingly solvating electrolytes restricts sulfur dissolution to near-surface regions. This controlled solubility promotes a mixed product state of sulfur and Li_2_S, resembling a solid-state-like conversion mechanism.

Time-resolved SAXS/WAXS shows an extended presence of Li_2_S during charge, overlapping with the reappearance of sulfur, a signature inconsistent with a pure dissolution–precipitation process. Sulfur is present in its amorphous form within the nanopores of the carbon black primary particles, and sulfur crystallizing at the outer surfaces of the particles is negligible. Composite discharge products consisting of nanocrystalline Li_2_S and amorphous Li_2_S_*x*_, as well as differences between the conversion during discharge and charge, underscore a discrepancy between equilibrium models explained by phase diagrams and kinetic processes in an electrochemical cell.

The SAXS invariant analysis indicates minimal electron density contrast changes, suggesting that active species remain confined within the electrode throughout cycling. Cryo-TEM combined with EELS provides direct real-space and chemical evidence for the simultaneous presence of Li_2_S and sulfur at 50% SOC, with sulfur consistently localized within the nanoporous carbon matrix. The observed asymmetry in sulfur/sulfide conversion during discharge and charge differs from pure equilibrium-based considerations, underscoring the importance of considering local kinetics and the presence of metastable phases when discussing conversion mechanisms in Li–S batteries.

Together, these observations establish that SPSEs facilitate a unique, spatially confined sulfur redox process that bypasses the drawbacks of highly solvating systems. SPSEs improve cycling stability by avoiding extended polysulfide diffusion to the anode, and they intrinsically allow for higher specific energy Li–S batteries by suppressing gelation at low E/S ratios. Given our finding of confined, amorphous sulfur in KB carbons, we propose that efficient QSS S/Li_2_S conversion should prioritize carbons with a substantial fraction of micro- and mesopores.

Crucially, this work demonstrates the power of integrating complementary techniques, combining ensemble-averaged structural information from operando scattering with localized chemical and structural information from cryo-TEM and EELS, to reveal complex electrochemical processes across length scales. This approach not only advances our understanding of QSS behavior in Li–S batteries but also provides a broadly applicable framework for decoding conversion-type reactions in next-generation energy storage systems.

## Supplementary Material



## Data Availability

All raw experimental data have been deposited on Zenodo and are accessible at DOI: 10.5281/zenodo.17144229

## Notes

Detailed experimental methods for electrode preparation, electrolytes, operando SAXS/WAXS, electrochemical testing, cryo-TEM, UV–vis, Raman, and BET analysis; descriptions of SAXS/WAXS fitting procedures and time-resolved scattering maps; supplementary figures on ternary phase diagrams of different electrolyte systems and electrochemical performance; digital images of separators collected from interrupted cells; UV–vis and Raman spectra of separators; particle size evolution; long-term cycling stability; additional cryo-TEM micrographs; and nitrogen sorption isotherms; discussion on time-resolved SAXS plots (PDF)
