# Supplementary material for: Bridging Solution and Solid-State Mechanism: Confined Quasi-Solid-State Conversion in Li–S Batteries
Source: ACS Energy Lett. 2025 Oct 25;10(11):5722–32. doi: 10.1021/acsenergylett.5c02093 (PMC12624839; doi:10.1021/acsenergylett.5c02093)
Supplement: Supplementary file 1 [file nz5c02093_si_001.pdf]

Supporting Information

Bridging Solution and Solid-State Mechanism: Confined Quasi-Solid-State Conversion in Li-S Batteries

Pronoy Dutta[1], Jean Marc von Mentlen[2], Soumyadip Mondal[3], Nikolaos Kostoglou[4,5], Bodo Wilts[1], Stefan A. Freunberger[3], Gregor A. Zickler[1], Christian Prehal[1*]

[1]Department of Chemistry and Physics of Materials, University of Salzburg, Jakob-Haringer-Straße 2A, 5020 Salzburg, Austria

[2]Department of Information Technology and Electrical Engineering, ETH Zürich, Gloriastrasse 35, 8092 Zürich, Switzerland

[3]Institute of Science and Technology Austria, Am Campus 1, 3400 Klosterneuburg, Austria

[4]Department of Materials Science, Montanuniversität Leoben, Franz-Josef-Straße 18, 8700, Leoben, Austria

[5]Institute of Geoenergy, Foundation for Research and Technology – Hellas, 73100 Chania, Greece

*Correspondence: christian.prehal@plus.ac.at

Experimental section:

Chemicals

Ketjenblack (KB) ECP-600JD conductive carbon black was purchased from Beyond Battery. Sulfur (99.98%), polytetrafluoroethylene dispersion (PTFE, 60 wt.% dispersion in water), lithium metal (99.9%), Lithium bis(trifluoromethanesulfonyl)imide (LiTFSI, anhydrous, 99.99%), 1,2- dimethoxy ethane (DME, anhydrous, 99.5%), Diethylene Glycol Dimethyl Ether (Diglyme, anhydrous, 99.5%) were purchased form Sigma Aldrich. 2H-tetrafluoroethyl 2,2,3,3-tetrafluoropropyl ether (HFE) was sourced from Apollo Scientific. The LiTFSI salt was dried at 120 °C for 24 h in vacuum while all the solvents were dried with molecular sieves (1.6 mm beads, 4Å, VWR chemicals) before using them as electrolytes.

Preparation of electrodes

Preparation of carbon/sulfur composite cathodes: All the cathodes in this study were prepared with a carbon black/sulfur composite having a carbon/sulfur ratio of 1:2 unless otherwise specified. The carbon/sulfur composite was prepared with a three-step melt-infiltration method. First, a specified amount of KB with sulfur powder, in a weight ratio of 1:2, was put in a 15 mL ball milling jar along with 3 mm ZrO_2 balls. The weight of the ZrO_2 balls was fixed at 10 times the weight of the mixture. The first ball milling step was performed at 500 rpm with 10 min of milling followed by 10 min of pause for a total of 10 repetitions. Once a homogenized mixture was ensured, the composite powder was then heated at 155 °C in a glass oven (Büchi, Switzerland), evacuated, and enclosed at 14 mbar for two hours to melt-infiltrate the sulfur into the pores of the KB. The resulting composite was again ball milled at 200 rpm for 10 min with a 10 min pause for 10 repetitions before using it for cathode preparation.

To prepare freestanding carbon/sulfur composite cathodes, the powder was mixed with dried PTFE prepared from a 60% aqueous dispersion of the binder. The aqueous PTFE was dried at 80 °C under vacuum overnight to ensure accurate measurement of binder in the freestanding cathode. 5 wt.% of the as-prepared dried PTFE powder was mixed with the composite in a mortar with ethanol as a dispersant. The resulting paste was then rolled manually with a stainless-steel rod over a glass plate to prepare the freestanding electrodes with a mean thickness of 80±10 μm. The electrodes were cut with an 8 mm puncher and dried overnight in ambient conditions before being used as cathodes in electrochemical cells.

Methods:

Electrochemical characterization

For the electrochemical characterizations, two different electrolytes were used. For the sparingly solvating electrolyte (SPSE), a 6M LiTFSI in 1,2-dimethoxyethane (DME) was prepared. The resulting solution was then added with HFE in a 1:2 volume ratio to reduce the overall viscosity of the electrolyte. The standard solvating electrolyte for operando studies was prepared with 1 M LiTFSI in diglyme (G2). For the GITT measurements, the solvating electrolyte was composed of DME/ 1,3-dioxolane (DOL) (v/v = 1:3) with 2 wt.% lithium nitrate ($LiNO_3$).

A custom-built split coin cell, assembled in an argon-filled glove box, was used for all the electrochemical measurements. The cathode was paired with a 12 mm aluminium current collector, one Whatman GF/A glass fiber, and one polyethylene (PE) (Targray, PE16A) stacked together as a separator and a 14 mm diameter Li metallic disk anode. 100 μL of electrolyte (corresponding to a relatively high

E/S ratio of 50 µL mg_S^{-1}) was used in all the electrochemical cells to ensure comparability between the different electrolyte types and cell setups used (operando SAXS/WAXS vs. cryo-TEM vs. standard testing setup). The cells were charged-discharged with galvanostatic cycling within a voltage range of 1.8 to 3V with Biologic SP-150 and VMP3 electrochemical workstation. The galvanostatic intermittent titration technique (GITT) was performed with a constant current pulse applied for 10 min following a relaxation step for 1 h.

Operando small and wide-angle X-ray characterization

Small-angle (SAXS) and wide-angle (WAXS) X-ray scattering measurements were performed with the Xenocs Xeuss 3.0 HR laboratory SAXS/WAXS system. For operando SAXS /WAXS, a Xenocs operando battery cell optimized for X-ray transmission through all the cell components was utilized. The Cu Kα X-rays emitted from Cu anode were introduced to the cathode through a 5 mm diamond window in the operando cell. The operando cell consisted of the same cell components as described above for the split coin cells. All the operando measurements were performed while the cells were galvanostatically cycled at C/10 with a Biologic SP150 electrochemical workstation. The SAXS and WAXS measurements were performed simultaneously with the SAXS detector set at a sample-to-detector distance of 400 mm and the WAXS detector set at position 4 as implemented by Xenocs. The 2D detector images were azimuthally averaged and corrected for transmission and empty cell contributions.

Data curation and preparation of time-resolved SAXS and WAXS maps

All scattering data were corrected for transmission and subsequently subtracted from the empty cell scattering. For the time-resolved SAXS intensity maps, all the data were normalized with the first cycle discharge (Figure 2,3) or with the SAXS intensity recorded before starting the discharge (i.e., at OCV). For the WAXS intensity maps, after empty cell correction, individual WAXS intensities were subtracted with 3rd order polynomial to highlight the corresponding changes with Li_2S and sulfur peak formation during discharging/charging.

The average intensity for a particular q range (q_1 to q_2) in SAXS was calculated following the equation:

$$\langle I \rangle = \int_{q_1}^{q_2} I(q)\ dq \qquad (SE1)$$

The crystallite size of Li_2S particles was calculated using the Scherrer equation $\tau = \frac{K\lambda}{\beta \cos(\theta)}$, where the shape factor (K) = 0.94, and Cu Kα wavelength (λ) = 0.154 nm was considered. Lorentzian peak fitting of individual Li_2S peaks was performed for the determination of peak width (β) and peak position (2θ). The area under the WAXS peaks was calculated using the Lorentzian peak area equation, $\pi \cdot I_0 \cdot \beta$ with I_0 being the amplitude of the fitted peak.

The scattering invariant ($\tilde{Q}$) was calculated using the equation,

$$\tilde{Q} = \int_{q_1}^{q_2} q^2 I(q)\ dq \qquad (SE2)$$

For all the particle size estimations from SAXS, mean q ($<q>$) was determined following the equation,

$$\langle q \rangle = \frac{\int_{q_1}^{q_2} q \cdot I(q)\, dq}{\int_{q_1}^{q_2} I(q)\, dq} \qquad (SE3)$$

Sample preparation and characterization for cryo-TEM

For characterization of the sample morphology and chemical composition by scanning transmission electron microscopy (STEM), a JEOL JEM-F200 transmission electron microscope operating at 200 kV was used. The microscope was equipped with a cold field emission electron source and a large, windowless JEOL Centurio EDX (Energy Dispersive X-ray emission) detector (100 mm^2, solid angle of 0.97 sr, and energy resolution below 133 eV@ MnKα), a CEOS CEFID energy filter, and two TVIPS XF416 CMOS cameras (pre- and postfilter). High-angle annular dark-field (HAADF) images, providing Z-contrast and EDX intensity maps, were obtained using a beam current of 0.1 nA and a beam diameter of 0.16 nm. Furthermore, cryogenic transmission electron microscopy/electron energy loss spectroscopy (Cryo-TEM/EELS) and cryo-scanning transmission electron microscopy/electron energy loss spectroscopy were used to investigate the elemental distribution of a post-mortem electrode. For all the analysis, the sample was kept below -170 °C in a MelBuild double tilt LN2 vacuum transfer or a GATAN Elsa cryo-TEM transfer holder.

To perform the cryo-TEM with cathodes free of any binder, 3 mg of the carbon/sulfur composite (33 wt.% sulfur) was added in 200 µL DME to prepare a homogenous colloidal mixture, which was then drop-casted on a glassy carbon (GC) disk (Sigradur G Discs, 16 mm diameter, 0.5 mm thickness) and dried overnight. After drying, the composite powder adhered to the GC disk, which was then assembled in the split coin cell as cathode with other components being the same as described before. The as-prepared cell was galvanostatically cycled to a certain state at C/20. After reaching the desired state of discharge/charge, the electrochemical cells were disassembled inside the glove box, and the composite powder was scratched off the smooth GC disk surface to a weighing paper. A 400 mesh Cu Quantifoil TEM grid, coated with a 10 nm thick amorphous holey carbon film, was then gently pressed over the cathode powder to make the sample stick to the carbon film. For 100% discharged cathodes, cryo measurements were performed with the MelBuild holder. In the argon gas-filled environment of the glove box, after loading the TEM grid in the MelBuild holder, the tip was retracted to preserve the inert atmosphere. Subsequently, the holder was first taken to a Gatan pumping station where the tip was extended at around 10^{-5} Torr and kept overnight. In the TEM, as the holder was inside the airlock, the tip was extended at a Pirani gauge current of 200 µA. The holder dewar was successfully filled with liquid nitrogen, and the tip temperature was set to be controlled at -160 °C. After an hour of cooling, the dewar was purged with helium to eliminate any potential bubble formation in the dewar, which would otherwise introduce vibrations during characterization. The helium bubbling drastically reduces the sample drift. The liquid nitrogen was replenished during the characterization when the dewar temperature rose above -155 °C, and the helium step was repeated.

However, this technique could not be applied to the 50% SOC and 100% SOC electrodes, as the tiny amounts of sulfur present in the cathode would evaporate in the TEM airlock or the pumping station. To maximize the stability of sulfur under high vacuum conditions, it is essential to take the sulfur-containing cathodes to cryogenic temperatures before exposing them to low-pressure conditions. To attain this, the Gatan Elsa cryo transfer holder was used. The prepared TEM grid was first loaded in a sealed transfer holder, which was used to transfer the grid from the glovebox to a liquid nitrogen-filled chamber placed right next to the glovebox antechamber to preserve the sample from possible air contamination. The transfer of the TEM grid to the Gatan holder was performed carefully under liquid nitrogen to ensure frost-free loading of these samples in the TEM.

Sample preparation and characterization for UV-Vis and Raman

To perform UV–Vis reflectance measurements and Raman spectroscopy, several coin cells were assembled and cycled to specific states of charge (SOC) and discharge (SOD). After cycling, the cells were disassembled inside an argon-filled glovebox, and the glass fiber separators were carefully collected for analysis. Solid-state UV-Vis measurements were conducted entirely within the glovebox using an Avantes AvaSpec-HSC 1024x58TEC-EVO (215 nm to 982 nm) spectrometer, coupled with an AVALIGHT-DH-S-BAL light source and fibre-optic probe system. The separators were positioned under an integrating sphere (AVASPHERE-50-REFL), and reflectance spectra were acquired with an integration time of 25 s and five accumulations per measurement. The raw reflectance data were smoothed using a Savitzky–Golay filter (second-order polynomial, 10-point window). All reflectance spectra were normalized to 1 for comparison.

For the Raman measurements, a piece of the separator was placed in a Linkam 16 mm, 0.1 mm coverslip and sealed with Kapton tape to prevent air exposure. The samples were then analyzed from the glass side, with the laser passing through the coverslip to probe the separator. The samples were analyzed using a Thermo Scientific DXR2 confocal Raman microscope equipped with a 532 nm, 10 mW laser using a 50 μm slit. Each spectrum was acquired with a 1s acquisition time over 150 frames to enhance the signal-to-noise ratio. The raw data were smoothed using a Savitzky–Golay filter (second-order polynomial, 20-point window).

Nitrogen adsorption–desorption measurements:

For the gas sorption measurements, three types of electrodes were prepared. First, activated carbon (KB) was mixed with 5 wt% PTFE to fabricate electrodes. Second, sulfur melt-infiltrated KB was also combined with 5 wt% PTFE to form electrodes. Finally, KB/S electrodes were cycled in cells with the SPSE electrolyte to full charge, after which the electrodes were recovered, washed with DME to remove residual salts, dried, and used for analysis.

The pore structure and surface characteristics were examined through nitrogen adsorption–desorption experiments carried out at –196 °C using a manometric gas sorption instrument (Autosorb iQ3; Anton-Paar QuantaTec, Boynton Beach, FL, USA). Ultra-high purity helium (99.999 %) was used to determine the void volume, while nitrogen of the same purity served as the adsorptive gas. Cooling during the measurements was achieved with a liquid nitrogen bath. Before analysis, the samples were controllably degassed under vacuum (~10^{-3} mbar) at 25 °C for 24 h to remove any physisorbed species. The specific surface area (SSA) was determined via the multi-point Brunauer–Emmet–Teller (BET) method, in accordance with the consistency criteria outlined in ISO 9277:2022.

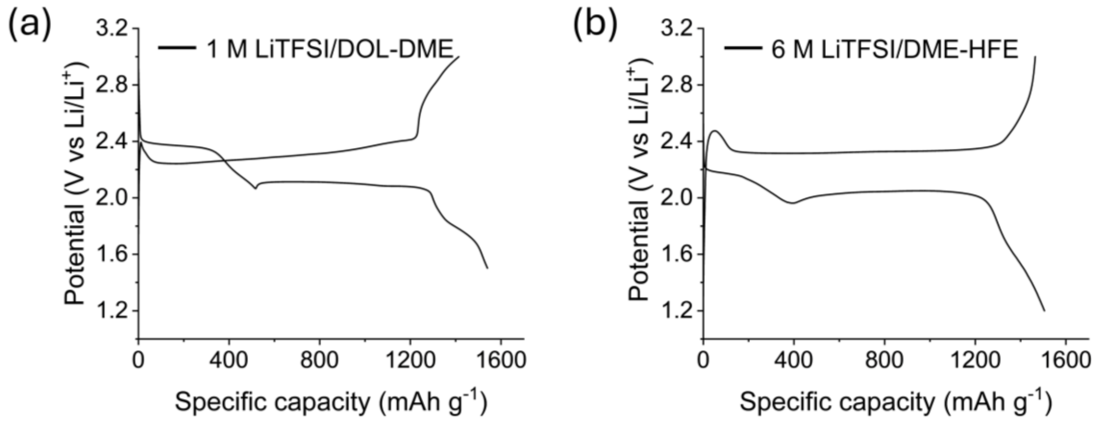

Figure S1: Galvanostatic discharging and charging for the Li-S battery cells with (a) standard solvating electrolyte (1 M LiTFSI in DOL-DME), and (b) sparingly solvating electrolyte (SPSE, 6 M LiTFSI in DME-HFE). Both cycles are performed at a rate of C/20. (1C = 1675 mAh g_s^{-1}).

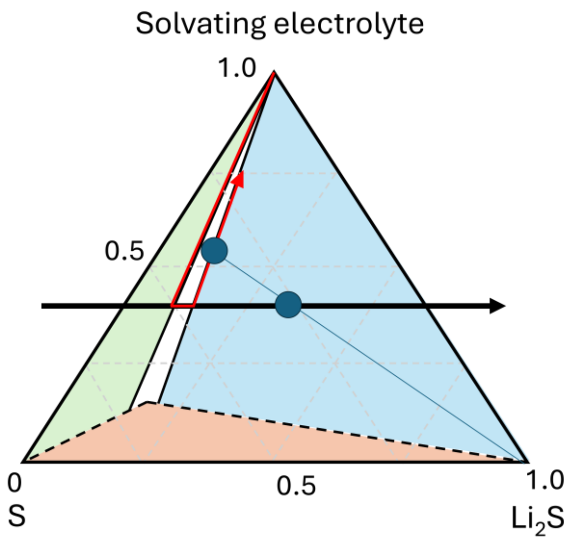

Figure S2: Ternary phase diagram of standard solvating electrolyte. The red line indicates the system's composition as the cathode is discharged and goes through different DODs.[1]

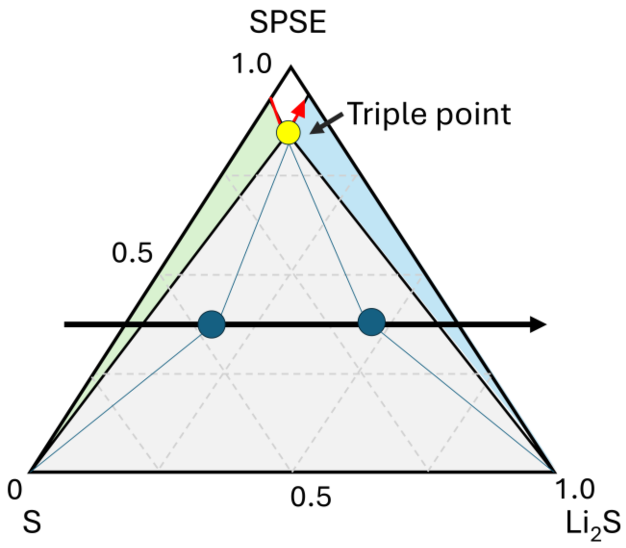

Figure S3: Ternary phase diagram of sparingly solvating electrolyte (SPSE) system with triple point formation.[1]The horizontal lines, parallel to the S-Li_2S edge, represent a system's intrinsic E/S ratio. The two blue circles indicate 50% and 75% discharge (from left), respectively. The blue circles also correspond to the system's "theoretical composition" at the respective states. However, the true solution composition at these points is determined by connecting a horizontal line through these system points to the phase boundary line (for solvating electrolytes in Figure S2) or to the triple point (for SPSEs in Figure S3). Therefore, as the system moves from lower DOD to higher DOD along a particular E/S line, it follows the phase boundaries as indicated by the red line in Figure S2. On the other hand, as indicated in Figure S3, the presence of the triple point takes the system to a prolonged three-phase equilibrium state, making almost the entire sulfur speciation lie in a particular equilibrium state, which is then reflected in the electrochemical discharge profile with the unchanged potential curve.

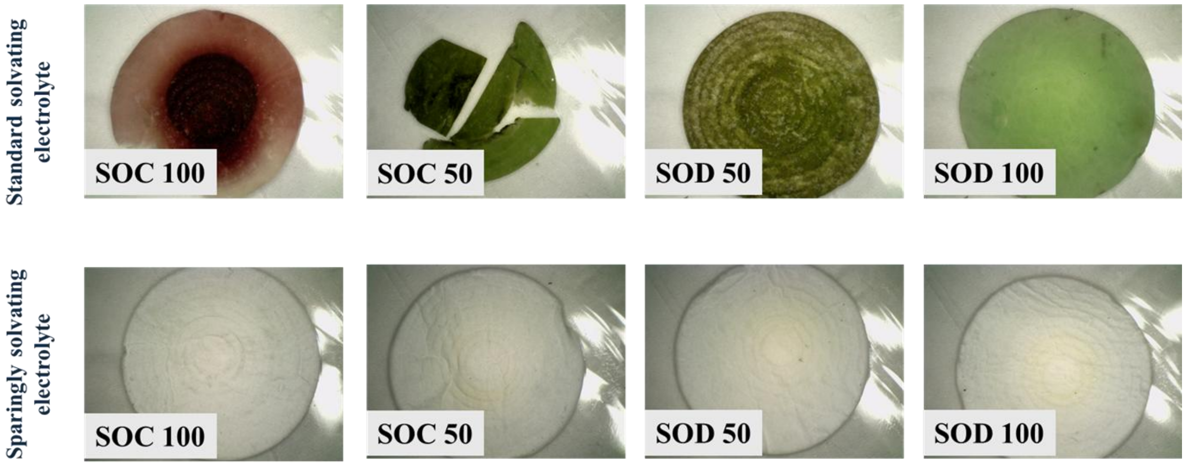

Figure S4: Digital images of the separators collected from batteries interrupted at different SOD/SOCs. The visible color changes in the standard solvating electrolyte system (upper panel) correspond to the evolving polysulfide chain lengths during cycling. The observed red-to-green color shift during discharge is consistent with the progressive reduction of higher-order polysulfides to shorter-chain species, corroborating the established understanding of sulfur conversion in ether-based electrolytes. In contrast, separators retrieved from sparingly solvating electrolyte (SPSE) cells exhibit a uniform pale-yellow color, independent of the SOD or SOC. This lack of visible color evolution suggests that soluble polysulfide formation is minimal, and the system remains in an equilibrium state throughout cycling.

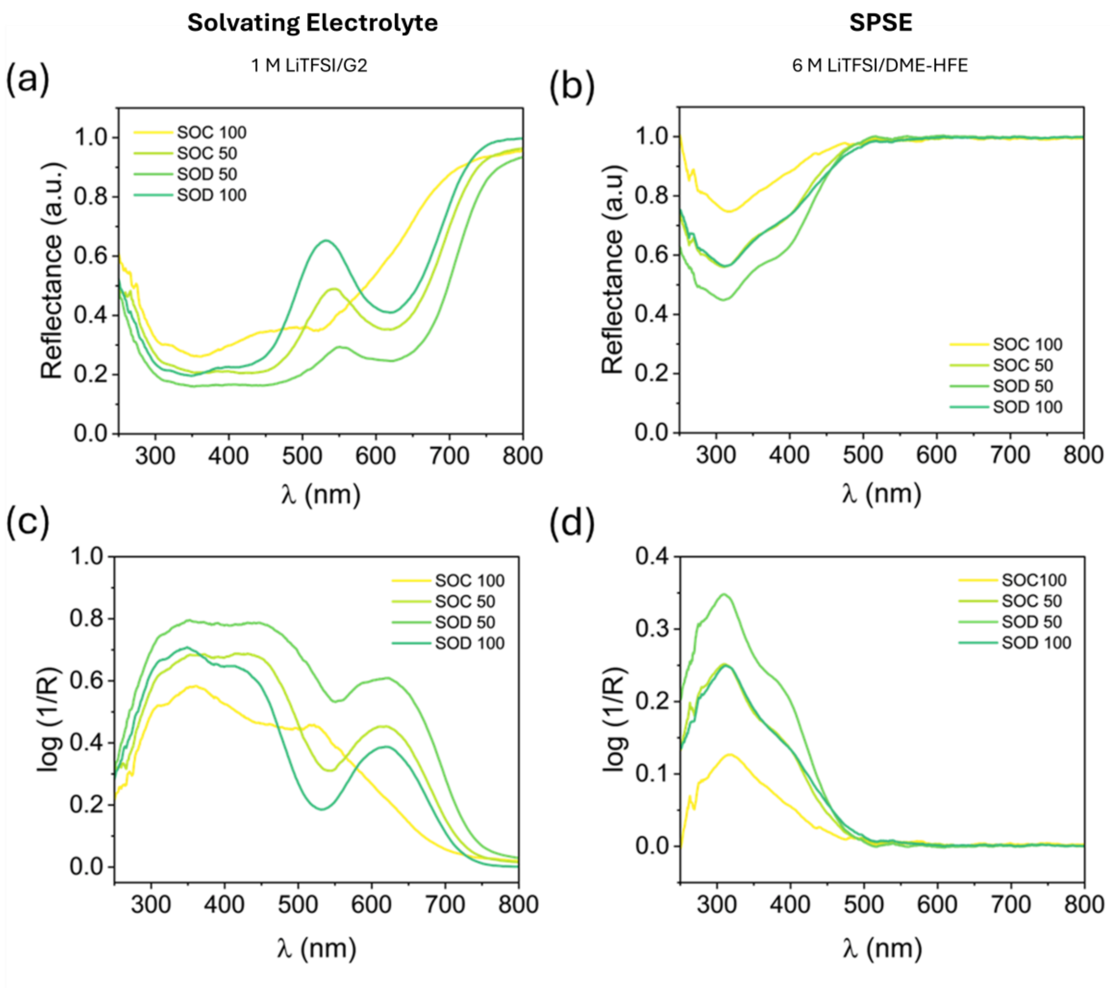

Figure S5: UV-Vis spectra of the separators collected from batteries with (a) standard solvating and (b) sparingly solvating electrolyte (SPSE). The corresponding absorbance calculated from reflectance is shown in (c) and (d). The separators from the standard electrolyte system display a systematic shift in reflectance from longer to shorter wavelengths as the cell moves from the fully charged to fully discharged state, consistent with the evolution of polysulfide chain lengths.[2,3] In contrast, SPSE-based separators exhibit no significant features in the 500–700 nm range, indicating negligible polysulfide presence. For the SPSE, the corresponding absorbance spectra calculated from the reflectance plot reveal a consistent peak at 309 nm, characteristic of short-chain polysulfides, suggesting that SPSE effectively suppresses the formation of longer-chain species.[4] For all UV–Vis analyses, control spectra were recorded from separators soaked only in the respective electrolytes and used as background for subtraction. Thus, the spectral features evolving at different states of discharge and charge can be attributed solely to polysulfide species in the system.

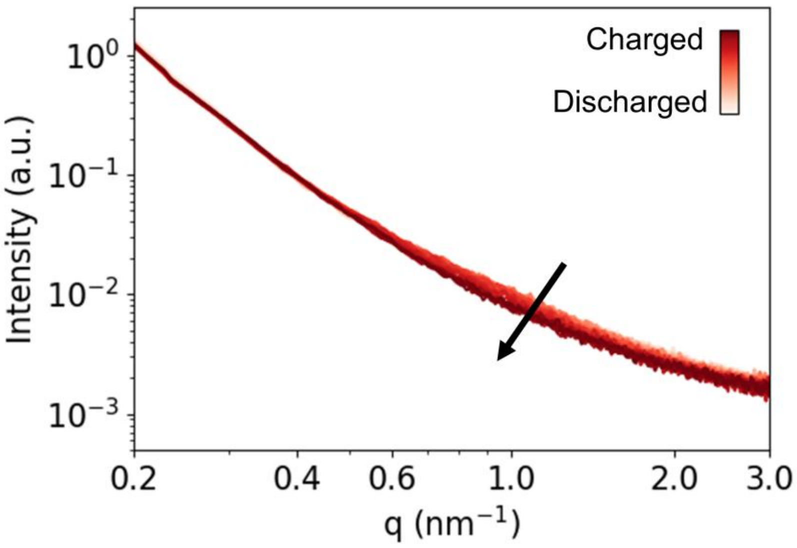

Figure S6: SAXS intensity plotted against scattering vector q during charging of the electrochemical cell with standard solvating electrolyte.

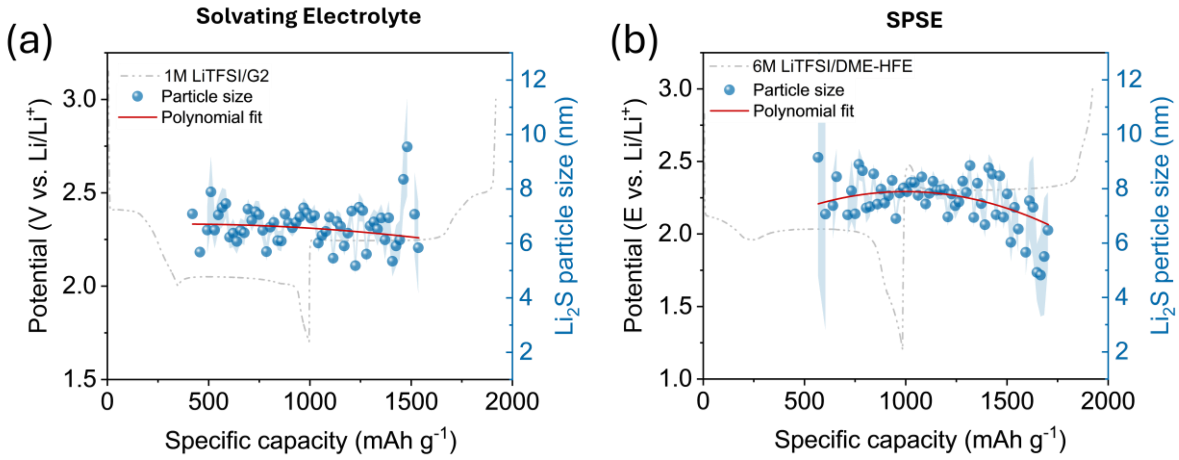

Figure S7: Time evaluation of Li_2S particle size during discharging/charging in (a) solvating electrolyte, and (b) sparingly solvating electrolyte (SPSE), estimated from Li_2S (111) WAXS peak fitting. Whereas the saturated electrolyte maintains an overall constant Li_2S size, the same shows a crystal size growth and shrinkage as evident from the fitted polynomial over the particle sizes. The blue shaded area indicates the error in size estimation arising from the Lorentzian peak fitting.

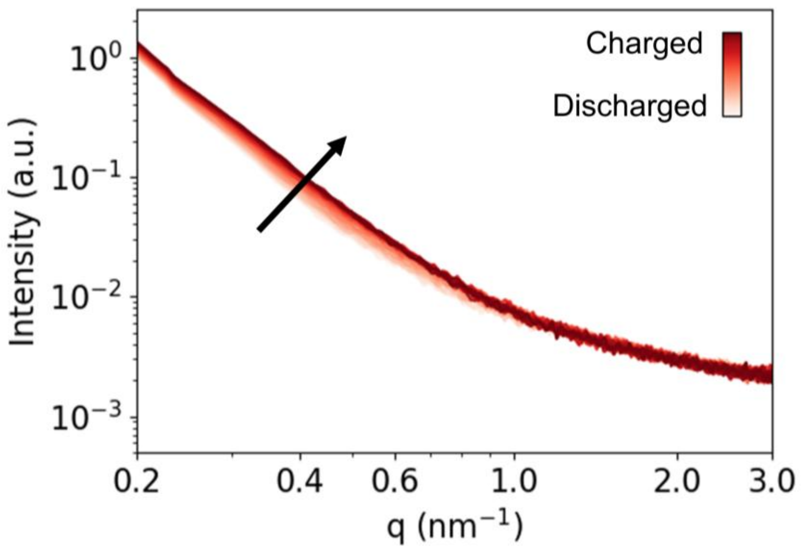

Figure S8: SAXS intensity versus scattering vector q during charging for the sparingly solvating electrolyte (SPSE). The arrow shows the region of significant activity during charging as the amorphous sulfur slowly forms.

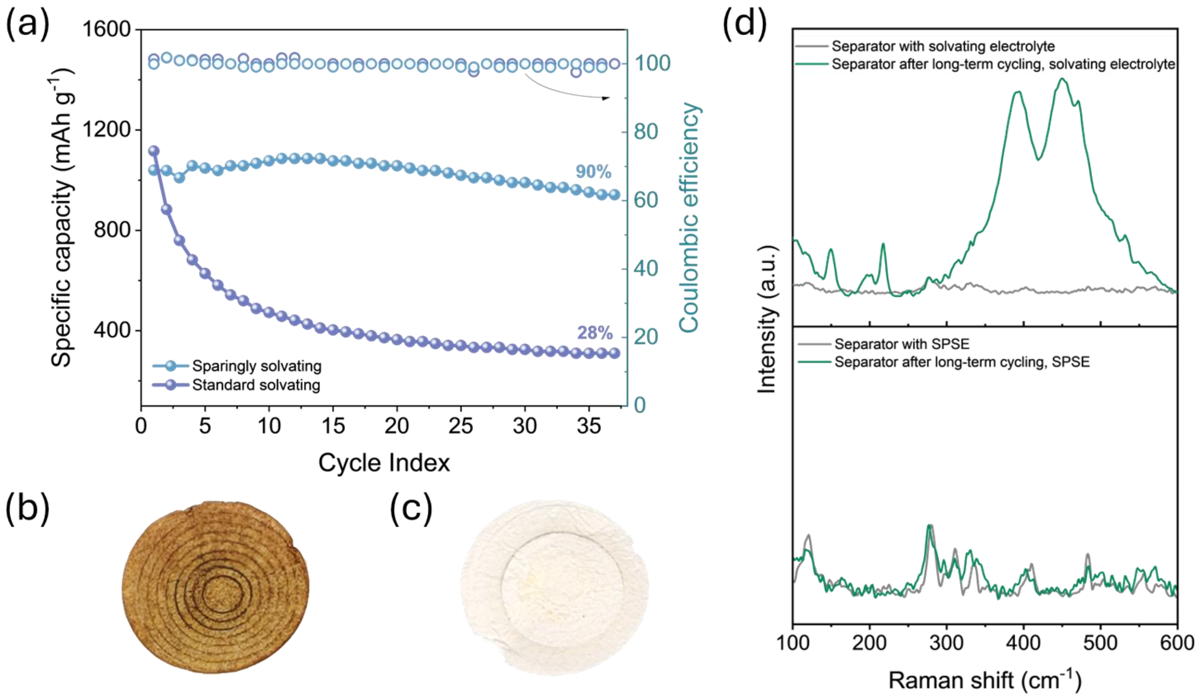

Figure S9: (a) Cycling stability of Li-S cells using standard solvating electrolyte and sparingly solvating electrolyte (SPSE), highlighting the contrast in capacity retention. Cycling was performed at a C/10 rate for 38 cycles. (b, c) Digital images of the separators retrieved after cycling from the solvating electrolyte cell (b) and the SPSE cell (c). (d) Comparative Raman spectra of the separators before and after long-term cycling. The top panel corresponds to the standard solvating electrolyte, and the bottom panel to the SPSE-based system. In each case, the cycled separator is compared with the respective electrolyte-soaked but uncycled separator.

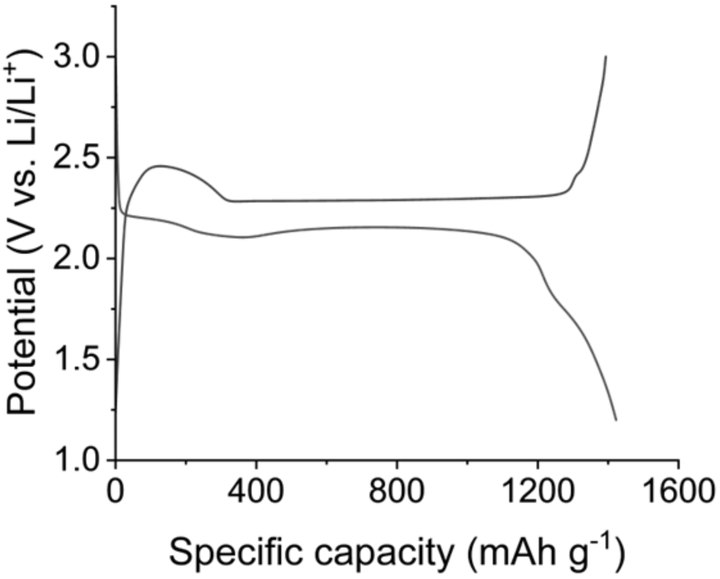

Figure S10: Galvanostatic discharging-charging in the sparingly solvating electrolyte (SPSE) with a cathode consisting of binder-free sulfur-infiltrated KB powder onto a glassy carbon disk; used for cryo-TEM measurements.

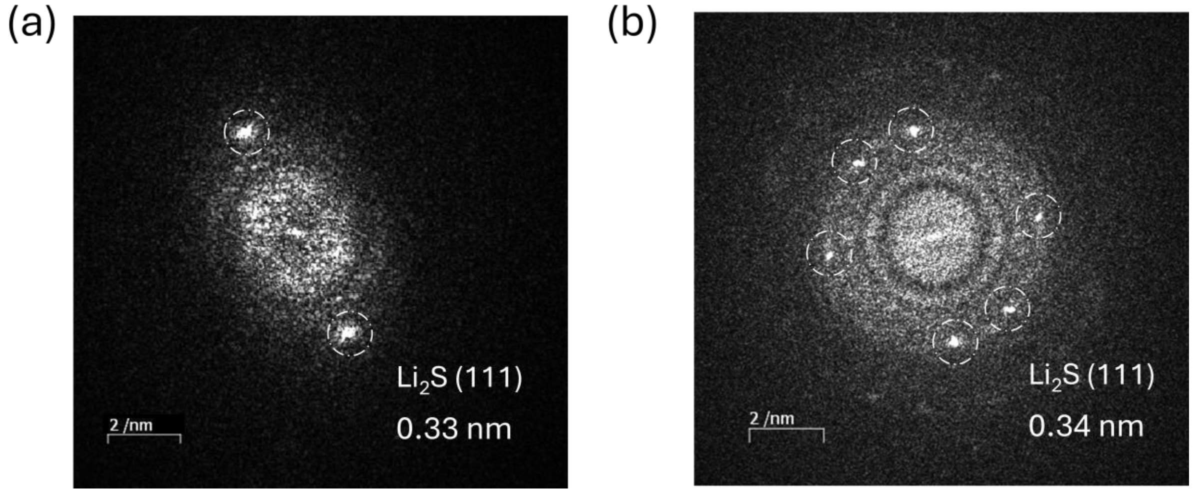

Figure S11: FFT of cryo-TEM images recorded at (a) 100% SOD and (b) 50% SOC. The calculation from FFT spots can be assigned to (111) planes of Li_2S for both 100% SOD and 50% SOC states.

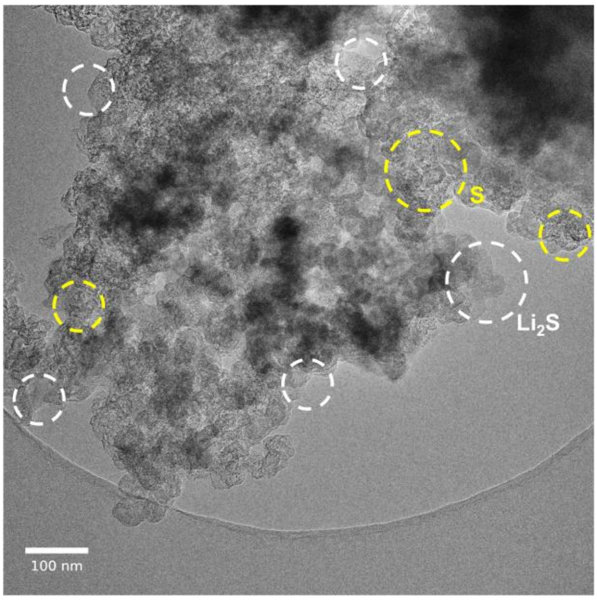

Figure S12: Cryo-TEM image of cathode powder at 50% SOC. The micrograph shows two distinct regions. The smoother surface on the edge shows crystallinity that resembles Li_2S. On the other hand, the rough, circular, aggregated structures reveal the presence of S with EELS with no sign of Li, confirming the formation of S at 50% SOC.

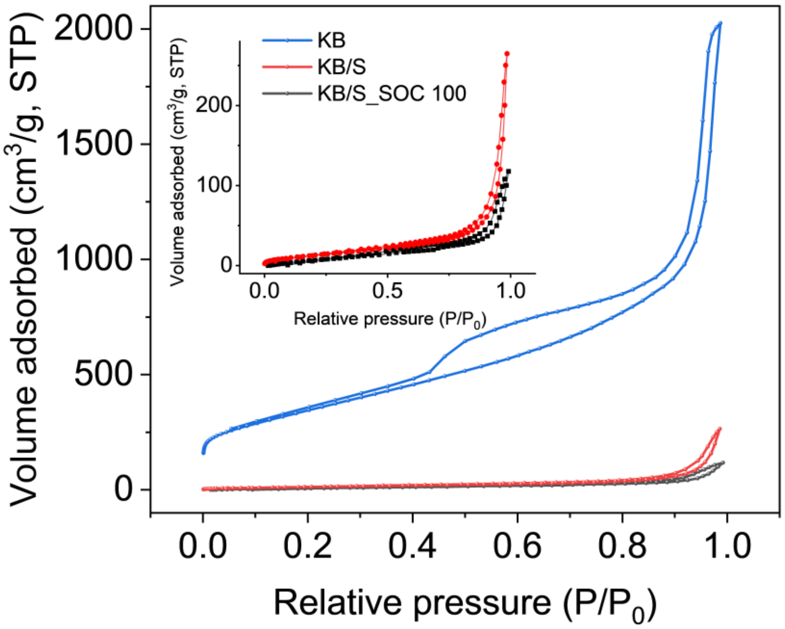

Figure S13: Nitrogen adsorption–desorption isotherms collected at 77 K for pristine KB, sulfur-infiltrated KB (KB/S), and KB/S electrodes fully charged with a sparingly solvating electrolyte (SPSE) cell. The pristine KB exhibits a Brunauer-Emmett-Teller (BET) surface area of 1269 $m^2 g^{-1}$, which decreases drastically to 54 $m^2 g^{-1}$ upon sulfur infiltration, indicating pore filling. After full electrochemical cycling with the SPSE, the BET surface area further decreases to 42 $m^2 g^{-1}$, consistent with additional sulfur redistribution inside the pores.

Supplementary Note 1: SAXS model fitting and simulation of sulfur-infiltrated KB particle

The SAXS data of the KB particles were modelled using a composite function incorporating contributions from aggregated structures and two distinct correlation regimes attributed to carbon domains and internal porosity. The total scattering intensity was expressed as the sum of a power-law term, describing large-scale fractal aggregates, and two Debye-Anderson-Brumberger (DAB)[7] terms representing density fluctuations on different length scales. The combined SAXS intensity model derived from these terms is,

$$I(q) = I_{Agg} + I_{PP} + I_{pore} \qquad (SE4)$$

$$I(q) = A\frac{1}{q^{n}} + \frac{\left(8\pi\, \Delta\rho_1{}^2\varphi_1(1 - \varphi_1)\xi_1{}^3\right)}{\left(1 + q^2\xi_1{}^2\right)^2} + \frac{\left(8\pi\, \Delta\rho_2{}^2\varphi_2(1 - \varphi_2)\xi_2{}^3\right)}{\left(1 + q^2\xi_2{}^2\right)^2} \qquad (SE5)$$

where q is the magnitude of the scattering vector, A is a scaling pre-factor, and n is the power-law exponent. $\Delta\rho_1$ and $\Delta\rho_2$ are the scattering length density (SLD) contrasts between the carbon matrix and air for the primary particle and pore domains, respectively. $\phi_1$and ϕ_2 represent the volume fractions of the primary particle network and internal pore domains, respectively. ξ_1 and ξ_2 are the corresponding correlation lengths characterizing the average size of structural heterogeneities in the primary particle network and pore domains.

After fitting the SAXS curve of the pristine KB particles, the sulfur-infiltrated KB system was simulated by adjusting the fitted scattering length density (SLD) and volume fractions of the structural components. As sulfur infiltrates the mesoporous structure, the effective SLD of KB particle increases significantly, while the SLD contrast between the carbon framework and the pore space diminishes due to the similar electron densities of carbon and sulfur. For instance, assuming 50% of the pore volume (with an overall porosity of 80%) is filled with sulfur, the effective SLD of the KB particle increases from 2.0×10^{9} cm^{-2} (empty) to approximately 6.4×10^{9} cm^{-2}, marking a 220% rise. Meanwhile, the SLD difference between the carbon walls and the sulfur-filled pores drops from 1.0×10^{10} cm^{-2} (for air-filled pores) to 1.9×10^{9} cm^{-2}, indicating a significant loss of internal contrast.

Supplementary Note 2: The time-resolved SAXS/WAXS maps

Time-resolved SAXS intensity profiles were normalized either to the first-discharge dataset or to the open-circuit-voltage (OCV) intensity response to accentuate subtle changes during cycling. Figure S11a presents data normalized to the discharge curve, whereas Figure S11b uses the OCV response. In Figure S11b, the onset of particle formation during discharge is reflected as an increased SAXS signal in the q_2 region (0.9-3 nm^{-1}), coinciding with Li_2S emergence in the WAXS pattern (Figure S11c). Upon charging, however, this SAXS feature shifts to lower q values as the Li_2S WAXS peaks gradually diminish, implying an increase in particle size despite Li_2S gradually depleting. We attribute this divergence to the concurrent presence of two discharge products, Li_2S_2 and Li_2S, rather than a single phase.[8] As the charging starts, the Li_2S oxidizes and contributes to more Li_2S_2 in the system, explaining the increased particle size observed in SAXS. Cryo-TEM (Fig. 4b) corroborates the existence of both species: the oxidized Li_2S_2 phase appears amorphous, accounting for its absence in WAXS.

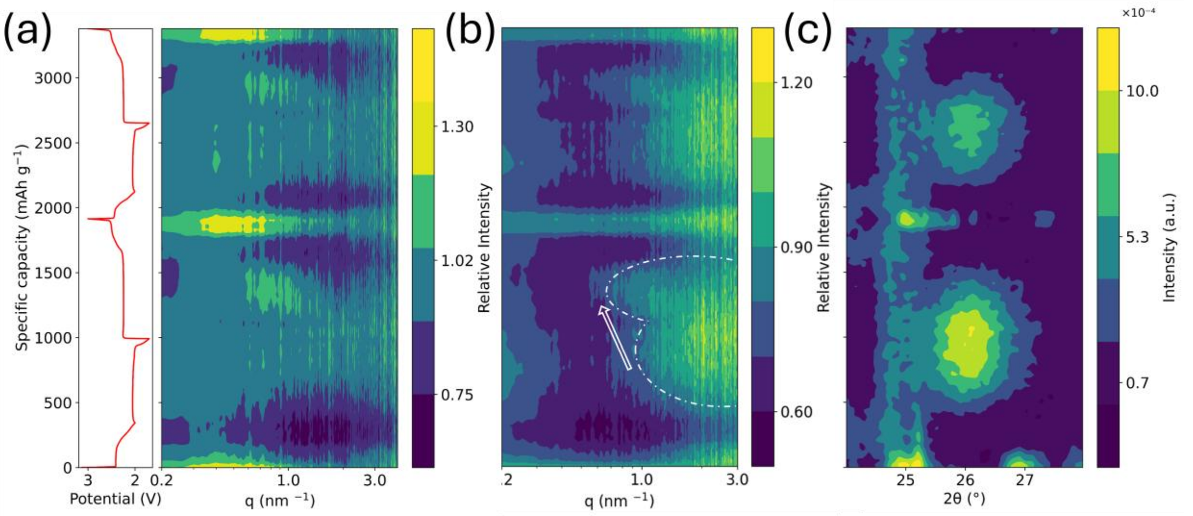

Figure S14: The time evolution of SAXS intensity versus scattering vector q as the solvating electrolyte cell is galvanostatically cycled for discharge/charge. Each SAXS curve in the plot is normalized with the (a) intensity dataset recorded at the end of first discharge and (b) at OCV to highlight the changes occurring in the high q region with the corresponding (c) WAXS scattering response.

References

(1) Song, Y.-W.; Shen, L.; Li, X.-Y.; Zhao, C.-X.; Zhou, J.; Li, B.-Q.; Huang, J.-Q.; Zhang, Q. Phase Equilibrium Thermodynamics of Lithium–Sulfur Batteries. *Nature Chemical Engineering* **2024**, *1* (9), 588–596. https://doi.org/10.1038/s44286-024-00115-4.

(2) Patel, M. U. M.; Demir-Cakan, R.; Morcrette, M.; Tarascon, J. M.; Gaberscek, M.; Dominko, R. Li-S Battery Analyzed by UV/Vis in Operando Mode. *ChemSusChem* **2013**, *6* (7), 1177–1181. https://doi.org/10.1002/CSSC.201300142;SUBPAGE:STRING:FULL.

(3) Patel, M. U. M.; Dominko, R. Application of In Operando UV/Vis Spectroscopy in Lithium–Sulfur Batteries. *ChemSusChem* **2014**, *7* (8), 2167–2175. https://doi.org/10.1002/cssc.201402215.

(4) Allison, A.; Davis, M. A.; Licht, F.; -, al; Logan, E. R.; Hebecker, H.; Ma, X. Operando Identification of Liquid Intermediates in Lithium–Sulfur Batteries via Transmission UV–Vis Spectroscopy. *J Electrochem Soc* **2020**, *167* (8), 080508. https://doi.org/10.1149/1945-7111/AB8645.

(5) Hagen, M.; Schiffels, P.; Hammer, M.; Dörfler, S.; Tübke, J.; Hoffmann, M. J.; Althues, H.; Kaskel, S. In-Situ Raman Investigation of Polysulfide Formation in Li-S Cells. *J Electrochem Soc* **2013**, *160* (8), A1205. https://doi.org/10.1149/2.045308JES.

(6) Wu, H. L.; Huff, L. A.; Gewirth, A. A. In Situ Raman Spectroscopy of Sulfur Speciation in Lithium–Sulfur Batteries. *ACS Appl Mater Interfaces* **2015**, *7* (3), 1709–1719. https://doi.org/10.1021/AM5072942.

(7) Debye, P.; Anderson, H. R.; Brumberger, H. Scattering by an Inhomogeneous Solid. II. The Correlation Function and Its Application. *J Appl Phys* **1957**, *28* (6), 679–683. https://doi.org/10.1063/1.1722830.

(8) Prehal, C.; von Mentlen, J.-M.; Drvarič Talian, S.; Vizintin, A.; Dominko, R.; Amenitsch, H.; Porcar, L.; Freunberger, S. A.; Wood, V. On the Nanoscale Structural Evolution of Solid Discharge Products in Lithium-Sulfur Batteries Using Operando Scattering. *Nat Commun* **2022**, *13* (1), 6326. https://doi.org/10.1038/s41467-022-33931-4.
